# Genetic variation in *CSF2* (5q31.1) is associated with longitudinal susceptibility to pediatric malaria, severe malarial anemia, and all-cause mortality in a high-burden malaria and HIV region of Kenya

**DOI:** 10.1186/s41182-022-00432-5

**Published:** 2022-06-25

**Authors:** Lily E. Kisia, Qiuying Cheng, Evans Raballah, Elly O. Munde, Benjamin H. McMahon, Nick W. Hengartner, John M. Ong’echa, Kiprotich Chelimo, Christophe G. Lambert, Collins Ouma, Prakasha Kempaiah, Douglas J. Perkins, Kristan A. Schneider, Samuel B. Anyona

**Affiliations:** 1 Department of Biomedical Sciences and Technology, School of Public Health and Community Development, Maseno University, Maseno, Kenya; 2 University of New Mexico-Kenya Global Health Programs, Kisumu, Siaya Kenya; 3 Center for Global Health, University of New Mexico, Albuquerque, NM USA; 4 Department of Medical Laboratory Sciences, School of Biomedical Sciences and Technology, Masinde Muliro University of Science and Technology, Kakamega, Kenya; 5 Department of Clinical Medicine, School of Health Sciences, Kirinyaga University, Kerugoya, Kenya; 6 Theoretical Biology and Biophysics Group, Theoretical Division, Los Alamos National Laboratory, Los Alamos, NM USA; 7 Centre for Global Health Research, Kenya Medical Research Institute, Kisumu, Kenya; 8 Department of Medicine, Loyola University Medical Center, Chicago, IL USA; 9 Department Applied Computer and Bio-Sciences, University of Applied Sciences Mittweida, Mittweida, Germany; 10 Department of Medical Biochemistry, School of Medicine, Maseno University, P.O. Box 333-40105, Maseno, Kenya

**Keywords:** *P. falciparum*, *CSF2*, GM-CSF, Malaria, Genotypes, Haplotypes, Diplotypes

## Abstract

*Plasmodium falciparum* infections remain among the leading causes of morbidity and mortality in holoendemic transmission areas. Located within region 5q31.1, the colony-stimulating factor 2 gene (*CSF2*) encodes granulocyte–macrophage colony-stimulating factor (GM-CSF), a hematopoietic growth factor that mediates host immune responses. Since the effect of *CSF2* variation on malaria pathogenesis remains unreported, we investigated the impact of two genetic variants in the 5q31.1 gene region flanking *CSF2*:g-7032 G > A (rs168681:G > A) and *CSF2*:g.64544T > C (rs246835:T > C) on the rate and timing of malaria and severe malarial anemia (SMA, Hb < 5.0 g/dL) episodes over 36 months of follow-up. Children (*n* = 1654, aged 2–70 months) were recruited from a holoendemic *P. falciparum* transmission area of western Kenya. Decreased incidence rate ratio (IRR) for malaria was conferred by inheritance of the *CSF2*:g.64544 TC genotype (*P* = 0.0277) and *CSF2* AC/GC diplotype (*P* = 0.0015). Increased IRR for malaria was observed in carriers of the *CSF2* AT/GC diplotype (*P* = 0.0237), while the inheritance of the *CSF2* AT haplotype increased the IRR for SMA (*P* = 0.0166). A model estimating the longitudinal risk of malaria showed decreased hazard rates among *CSF2* AC haplotype carriers (*P* = 0.0045). Investigation of all-cause mortality revealed that inheritance of the GA genotype at *CSF2*:g-7032 increased the risk of mortality (*P* = 0.0315). Higher risk of SMA and all-cause mortality were observed in younger children (*P* < 0.0001 and *P* = 0.0015), HIV-1(+) individuals (*P* < 0.0001 and *P* < 0.0001), and carriers of HbSS (*P* = 0.0342 and *P* = 0.0019). Results from this holoendemic *P. falciparum* area show that variation in gene region 5q31.1 influences susceptibility to malaria, SMA, and mortality, as does age, HIV-1 status, and inheritance of HbSS.

## Introduction

In 2020, the estimated number of malaria cases reported worldwide was 241 million [1]. A large proportion (95%; 228 million) of the cases occurred in the African region, mainly attributed to *Plasmodium falciparum* (*P. falciparum*) infections (> 99.8% of the total cases) [1]. Globally, there were ~ 627,000 malaria-related deaths, for which the most vulnerable population were children under 5 years of age [1]. Accordingly, 77% of the global malaria mortalities were children residing in the World Health Organization (WHO) African region [1]. In western Kenya, a region holoendemic for *P. falciparum* transmission, malaria is one of the primary causes of childhood morbidity and mortality [2, 3]. In this region, severe *P. falciparum* infections manifest as severe malarial anemia [SMA, hemoglobin (Hb) < 5.0 g/dL] [4, 5]. Our previous investigations in the study area show that HIV-1 significantly increases the cross-sectional risk of SMA [6].

The pathophysiology of SMA is complex, and includes the destruction of malaria parasite-infected and non-infected erythrocytes, as well as decreased erythropoiesis [7]. We have demonstrated that variability in genes that encode immune-response proteins plays a key role in the pathogenesis of SMA, largely through imparting changes in soluble mediators of inflammation [8]. However, the causal molecular basis of the disease has not been fully elucidated.

Granulocyte monocyte-colony stimulating factor (GM-CSF) is a hematopoietic growth factor that facilitates the differentiation of progenitor cells into three lineages in the bone marrow, namely the lymphoid, myeloid, and erythroid progenies [9–11]. GM-CSF has been shown to promote growth and differentiation of leucocytes, and enhance release of other cytokines, which are central mediators of host immune responses [12, 13]. GM-CSF is secreted by an array of cell types including mast cells, B cells, activated T cells, fibroblasts, macrophages, vascular endothelial cells, and various oncogenic cells [9]. Secretion of GM-CSF is often accompanied by the release of additional inflammatory mediators, such as the granulocyte colony-stimulating factor (G-CSF) [10, 12], which in turn, modulate GM-CSF production through feedback regulatory mechanisms [14, 15].

The importance of GM-CSF in host immune response to both non-infectious and infectious diseases has been reported for tumor growth and metastasis, Crohn’s disease (CD), and tuberculosis [16–18]. With regard to malaria, studies utilizing murine models have reported: (1) a reduction in the levels of erythropoietic-related cytokines, including GM-CSF; (2) a negative correlation between GM-CSF concentrations and enhanced pathology in malarial anemia; and (3) elevated levels of GM-CSF in lethal malaria [19, 20]. In the context of human malaria, the toll-like receptors (TLR) 7/8 stimulated production of GM-CSF was elevated in cord blood cells of infants with evidence of past placental malaria [21], suggesting a profound effect on the fetal immune system, with the differential alternations in innate immune responses predicting the risk of malaria during the first year of life [21]. Moreover, elevated serum levels of GM-CSF have been reported in cases of severe *P. falciparum* malaria [22]. Previous investigations in our laboratories identified elevated levels of GM-CSF in children with SMA compared to those with non-SMA, and elevated GM-CSF levels in children with *P. falciparum* and HIV-1 co-infection relative to children with malaria alone [23, 24].

The gene that encodes GM-CSF is colony-stimulating factor 2 (*CSF2*), which is located on the human chromosome at 5q23-31, in close proximity to interleukin 3 *(IL3)* within a T helper type 2-associated gene cluster [25]. *CSF2* spans ~ 2.5 kb in length, and encompasses 4 exons and 3 introns [26]. *CSF2* expression is regulated at both the transcriptional and post-transcriptional stages [27–29]. Elements in the proximal promoter of *CSF2* were shown to contribute to transcriptional regulation of *CSF2* by multiple transcription factors such as the AP-1, ZEB1, NF-AT, and GATA [27–29]. At the post-transcriptional regulation level, a stretch of AU-rich elements (ARE) in the 3′ untranslated region (3′-UTR) of *CSF2* mRNA mediates the binding of ARE-binding proteins to *CSF2* mRNA, causing degradation of this transcript [28, 29]. Interestingly, various distal enhancers were shown to modulate transcription of human target genes such as *CCR5*, *BRN3A*, *CDKN1C, VEGFA*, *ADGB, C-myb, SOX-9, SCNA, C-myc,* cPLA2α gene (*PLA2G4A*) and renin gene (*REN*) by forming cognate enhancer–promoter loops with the target gene promoter site [30–36]*.* Some of these distal enhancers are located at varied distances upstream of the transcription start site (TSS) of the corresponding gene: ~ 552 kb for *CCR5*, ~ 55 kb for *BRN3A*, over 100 kb for *CDKN1C,* ~ 157 kb for *VEGFA*, ~ 34 kb for *C-myb,* ~ 635 kb for *SOX-9,* ~ 9.5 kb for PLA2G4A, and ~ 5.3 kb for *REN* [31, 32, 34, 35]*.* In contrast, other distal enhancers are located at varied distances downstream of corresponding TSS of the gene: 216 kb for *ADGB,* 85 kb for *SCNA,* and 1.43 Mb for *C-myc* [33, 36]. Collectively, these studies show that the enhancers can be located either up- or downstream at varied distances of target genes.

A number of studies have found associations between polymorphic variability in the *CSF2* gene and outcomes of various disease [37–42], while other studies observed no relationships [43, 44]. In order to understand the influence of *CSF2* variants on susceptibility to malaria and SMA, the current study investigated the impact of two SNPs flanking the *CSF2:*g.-7032 G > A (rs168681:G > A) and *CSF2:*g.64544T > C (rs246835:T > C). The first SNP, rs168681:G > A is about 6 kb upstream of the 1 kb proximal promoter of *CSF2* and has recently emerged among the genetic biomarkers for prediction of the urinary nicotine metabolite ratio in multiethnic samples [45]. The second SNP, rs246835:T > C has not been linked to any diseases. We selected these two SNPs based on their characteristics of (1) minor allele frequencies (MAF) ≥ 10% in African populations and (2) the ability to impart functional changes in transcription-factor binding sites (TFBS) within corresponding distal enhancers [27, 28]. The relationship between the SNPs (and their haplotypes and diplotypes) on susceptibility to malaria, SMA and mortality were investigated longitudinally over a 36-month follow-up in a cohort of children (*n* = 1654; age 2–70 months at enrollment) resident in a *P. falciparum* holoendemic region of Kenya.

## Methods

### Study area

This study was conducted at Siaya County Referral Hospital (SCRH), located in a holoendemic *P. falciparum* transmission area in western Kenya. Details of the study site have previously been published [46]. One of the primary causes of childhood mortality and morbidity in the Siaya community is *P. falciparum* malaria [47], whose transmission has remained stable, despite malaria control efforts [48]. SMA accounts for one of the highest percentages of hospital-bed occupancies in SCRH, and results in significant in-hospital morbidity and mortality [4]. Individuals inhabiting the study area are predominantly from the Luo ethnic group (> 96%), a culturally homogeneous population [49].

### Study participants

Children presenting with suspected malaria infections and those reporting for routine vaccinations were recruited at SCRH. After screening for malaria parasites, children (aged 2–70 months) with varying severities of malarial anemia (*n* = 1319) and aparasitemic controls (*n* = 335) were enrolled. Children were excluded from the study if they presented with non-*falciparum* parasite strains, had confirmed cerebral malaria, were previously hospitalized for any reason, or had reported use of antimalarial therapy in the two preceding weeks. The current study utilized two cohorts recruited and followed with identical parameters across a temporal continuum: cohort 1 (2003–2005; *n* = 777) and cohort 2 (2007–2012; *n* = 877).

### Longitudinal follow-ups

After enrollment (Day 0), children (*n* = 1654) were scheduled for follow-up visits on day 14 (if they were febrile upon enrollment) and quarterly over 36 months [50–52]. Parents/guardians who failed to return for scheduled quarterly follow-up visits were traced by the study team at their residence to check the child’s health status, including mortality. Each residence was identified by a GIS/GPS surveillance system. In addition, parents/guardians were asked to return to the hospital any time their child was febrile [acute febrile episode(s)]. Physical evaluations and laboratory tests required for comprehensive clinical management of the patients were performed at enrollment, day 14, and each acute and quarterly visit (complete blood counts [CBC], malaria parasitemia measures, and evaluation of bacteremia where indicated). For all acute episodes and scheduled visits, children were managed according to the Ministry of Health-Kenya guidelines.

### Laboratory procedures

Heel and/or finger-prick blood samples (< 100 µL) and venipuncture blood (1–3 mL) were obtained and used to determine parasite densities. Giemsa-stained thin blood smears were prepared, examined, and asexual malaria parasite densities determined as previously published [46]. Complete blood counts (CBCs) were determined using a Beckman-Coulter AcT diff2 (Beckman-Coulter Corporation, Brea, CA, USA). At enrollment, based on Hb concentrations, children with any density parasitemia, were stratified into either non-SMA (Hb ≥ 5.0 g/dL; *n* = 1029) or SMA (Hb < 5.0 g/dL; *n* = 290), according to the WHO definition of SMA [53]. Aparasitemic children (*n* = 335; *P. falciparum* negative blood smear), recruited from vaccination clinics at SCRH, were age and gender-matched.

Since co-infections influence the severity of malarial anemia in Siaya, all children were tested for HIV-1 and bacterial infections as previously described [6, 54]. Parents/legal guardians of participating children received pre- and post-test HIV&AIDS counseling. At the time of enrollment, none of the children were on antiretroviral therapy for HIV-1.

To further discern chronic anemia resulting from genetic factors, sickle cell traits and α^3.7^-thalassemia deletions were investigated. Sickle cell status was determined using the alkaline cellulose acetate electrophoresis on Titan III plates (Helena BioSciences, Sunderland, UK). The α^3.7^-thalassemia deletion variants were determined as previously described [55].

### CSF2 genotyping

The BuccalAmp™ DNA extraction kit (Epicentre Biotechnologies, Madison, WI, USA) was used to extract genomic DNA from buccal swabs collected from each of the 1654 study participants described above. The resulting genomic DNA was then amplified using the Genomiphi DNA amplification kit (GE Healthcare, Life Sciences, Marlborough, MA, USA), according to the manufacturer’s instructions. Genetic variants rs168681:G > A and rs246835:T > C were genotyped using the TaqMan 5′ allelic discrimination Assay-By-Design (assay IDs C_3285157_20 and C_2397167_10, respectively). Allelic discrimination was performed using the StepOne™ Software Version 2.3 (Thermo Fisher Scientific, Carlsbad, CA, USA).

Amplification was conducted in a total reaction volume of 20 µL, with the following conditions: initial denaturation at 60 °C for 30 s and 95 °C for 10 min., followed by 40 cycles of 95 °C for 15 s and 60 °C for 1 min., and a final extension (60 °C for 30 s). To validate results obtained with the Taqman^®^ real-time genotyping assays, 10% of the samples were randomly selected and genotyped using restriction fragment length polymorphism PCR. There was 100% agreement between the two methods.

### Data analyses

Data were analyzed using R version 3.1.4 [56]. Data from both cohorts (1: 2003–2005, *n* = 777, and 2: 2007–2012, *n* = 877) were combined into one data set. However, cohort was kept as a categorical covariate to account for potential changing malaria incidence across time. Metric variables upon enrollment were compared across aparasitemic, non-SMA, and SMA patients. For metric variables, boxed plots, histograms, and Q–Q-plots were used to identify normally distributed variables. The median (interquartile range) and mean (standard deviation) were calculated for each variable. For normally distributed variables, one-way ANOVAs and two-sample *T*-tests were used. If normality was violated, Kruskal–Wallis tests and Mann–Whitney *U* tests were used. The distribution of categorical variables was compared between the groups (aparasitemic, non-SMA, and SMA) using a Chi-square test for homogeneity. If cell count was < 20, a generalized Fisher’s exact test was performed. If numerically not feasible, *P*-values were approximated using a Monte Carlo method, with *B* = 50,000 contingency tables.

Haplotypes and diplotypes were constructed to investigate the effects of the genetic architecture determined by the *CSF2:*g.-7032 G > A (rs168681:G > A) and *CSF2:*g.64544T > C (rs246835:T > C) loci. The top-level was the individual diplotypes, consisting of 2 of the 4 possible haplotypes; GT, GC, AT, and AC. At the marginal levels, the diplotypes gave rise to the (1-locus) genotypes at *CSF2:*g.-7032 G > A (GG, GA, AA) and *CSF2:*g.64544T > C (TT, TC, CC). For all generalized linear and hazard models performed, the effects of the genetic architecture were coded as 0/1 variables that reflected: (1) per locus contributions determined by the mutant homozygotes and heterozygotes (indicating additive and dominant effects), (2) haplotype contributions (reflecting epistatic interactions), and (3) diplotype contributions (reflecting positional effects). The respective wild types were subsumed as a baseline factor. Hence, we coded the following 0/1 variables: (1) genotypes GA, AA for *CSF2*:g.-7032 G > A and TC, CC for *CSF2*:g.64544T > C; (2) haplotypes GC, AT, AC; and (3) diplotypes GT/GC, GT/AT, GT/AC, GC/GC, GC/AC, AT/AT, AT/AC, AC/AC. We tested for differences in the frequency distributions between the aparasitemic, non-SMA, and SMA groups with the generalized Fisher’s exact test with simulated *P*-values. Exact tests for deviations from Hardy–Weinberg equilibrium (HWE) at the rs168681:G > A and the rs246835:T > C loci were performed as previously described [57]. Furthermore, due to low cell counts for some genotypes, Fisher’s exact test was performed to estimate departures from linkage-equilibrium between the two SNPs. Linkage disequilibrium (LD) of these two SNPs was determined by HaploView (version 4.2, Broad Institute, Cambridge, MA, USA).

The effect of covariates on the rate of malaria and SMA episodes was investigated using Poisson regression, with the model of best fit determined using Akaike’s information criterion (AIC). *CSF2* genetic variants, age, sex, cohort, hemoglobinopathies (sickle cell traits and α^3.7^-thalassemia deletions), and co-infections (HIV-1 and bacteremia) were included as covariates. Only co-variables present in more than two samples were retained in the model.

An ordered multiple-outcome-per-subject Cox proportional hazard model was used to investigate the longitudinal influence of covariates (as listed above) on the occurrence of malaria and SMA episodes. Independent increments were used according to Anderson–Gill and data were right-censored to account for cases in which a patient was malaria or SMA free upon clinical presentation.

The influence of covariates on the risk of mortality was investigated using a Cox proportional hazard model. For each patient, either mortality or survival occurred at the last hospital visit. The last recorded hospital visit was the time to the event, or censoring, in the case of survival. Age (at the last hospital visit) was included as a covariate. The model was fit using all-cause mortality as the outcome variable.

## Results

### Demographic, clinical, and laboratory characteristics

Children (*n* = 1654) were categorized into aparasitemic (*n* = 335), and parasitemic (*n* = 1319) stratified into non-severe malarial anemia (non-SMA, hemoglobin; Hb ≥ 5.0 g/dL, *n* = 1029), and severe malarial anemia (SMA, Hb < 5.0 g/dL, *n* = 290) groups. The demographic, clinical, and laboratory characteristics of study participants are presented in Table 1. The distribution of sex was comparable (*P* = 0.6991) across the groups. Although the distribution of SMA cases were equal in cohort 1 (2003–2005) and cohort 2 (2007–2012), they differed significantly between aparasitemic and non-SMA groups (*P* = 5.5311 × 10^–11^). Children with SMA were younger (*P* = 0.0152) relative to aparasitemic and non-SMA groups, but the difference was not significant after correction for multiple testing. Axillary temperature differed across the groups and was higher in children with non-SMA (*P* = 1.4420 × 10^–14^), compared to those with parasitemia or SMA. Children were stratified a priori based on Hb concentrations. As such, relative to both the aparasitemic and non-SMA groups, children with SMA presented with significantly decreased hematocrit levels (*P* = 1.4732 × 10^–174^). White blood cell counts progressively increased across the groups (*P* = 4.3233 × 10^–12^), as did the mean corpuscular volume (*P* = 7.9395 × 10^–21^), with the SMA group having the highest levels (Table 1). Parasite densities were lower in children with SMA relative to non-SMA (*P* = 2.9626 × 10^–25^). The presence of HIV-1 did not significantly differ across groups (*P* = 0.0729), nor did bacteremia status (*P* = 0.1583). However, the distribution of sickle cell genotypes differed across groups (*P* = 1.9990 × 10^–06^), with a lower frequency of HbAS carriage in children with non-SMA and lowest frequencies among children with SMA. The distribution of α^3.7^-thalassemia deletion variants were also different across the groups (*P* = 0.0433), with the highest frequencies of single and double-deletions in children SMA, however the difference is not significant after correction for multiple testing.

**Table 1:** Demographic, clinical, and laboratory characteristics of the study participants

Characteristics	*N*	Total	Aparasitemic (MPS negative)	Non-SMA (Hb ≥ 5.0 g/dL)	SMA (Hb < 5.0 g/dL)	*P*
Number of participants		1654	335	1029	290	
Sex [*n*, (%)]						
Female	1654	822 (49.70)	168 (50.15)	504 (48.98)	150 (51.72)	0.6991^a^
Male		832 (50.30)	167 (49.85)	525 (51.02)	140 (48.28)	
Study cohort [*n*, (%)]						
One (1)	1654	777 (46.98)	209 (62.39)	423 (41.11)	145 (50.00)	5.5311 × 10^–11a^
Two (2)		877 (53.02)	126 (37.61)	606 (58.89)	145 (50.00)	
Age, months	1651	13.85 (8.14)	13.82 (8.67)	14.20 (7.88)	12.64 (8.34)	0.0152^b^
Axillary temperature, °C	1636	37.67 (1.06)	37.27 (1.00)	37.80 (1.09)	37.69 (0.89)	1.442 × 10^–14a^
Hematological and parasitological parameters						
Hemoglobin, g/dL	1639	7.47 (2.51)	9.40 (2.63)	7.79 (1.82)	4.13 (0.73)	NA
Hematocrit, %	1609	24.34 (7.72)	29.52 (80)	25.40 (5.76)	14.22 (3.88)	1.4732 × 10^–174b^
White blood cells, × 10^3^/µL	1602	13.40 (6.98)	13.06 (8.11)	12.77 (5.52)	16.16 (9.39)	4.3233 × 10^–12b^
Mean corpuscular volume, fL	1603	70.10 (9.30)	69.87 (8.41)	68.89 (8.48)	74.90 (11.46)	7.9395 × 10^–21b^
Parasite density, MPS/µL	1644	55,716.67 (111,089.67)	0.00 (0.00)	72,983.96 (124,507.30)	59,082.88 (103,946.74)	2.9626 × 10^–25b^
Endemic co-infections						
HIV1, [*n* (%)]						
Negative (0)	1641	1573 (95.86)	315 (96.04)	990 (96.49)	268 (93.38)	0.0729^c^
Positive (1)		68 (4.14)	13 (3.96)	36 (3.51)	19 (6.62)	
Bacteremia						
Negative (0)	1639	1518 (92.62)	295 (90.77)	960 (93.57)	263 (91.32)	0.1583^a^
Positive (1)		121 (7.38)	30 (9.23)	66 (6.43)	25 (8.68)	
Genetic variants						
Sickle cell trait, *n* (%)						
Hb AA	1619	1358 (83.88)	252 (78.26)	848 (83.38)	258 (92.14)	1.9990 × 10^–06d^
Hb AS		243 (15.01)	60 (18.63)	165 (16.22)	18 (6.43)	
Hb SS		18 (1.11)	10 (3.11)	4 (0.39)	4 (1.43)	
α^+^-Thalassemia deletion, *n* (%)						
αα/αα	1405	596 (42.42)	120 (43.96)	375 (42.86)	101 (39.30)	0.0433^a^
-α^3.7^/αα		526 (37.44)	84 (30.77)	335 (38.29)	107 (41.63)	
-α^3.7^/-α^3.7^		283 (20.14)	69 (25.27)	165 (18.86)	49 (19.07)	

Data are presented as mean (standard deviation). Children (*n* = 1654) were categorized into aparasitemic controls (*n* = 335; no MPS) and according to the WHO definition of SMA [53], into either non-SMA (*n* = 1029; Hb ≥ 5.0 g/dL) or SMA (*n* = 290; Hb < 5.0 g/dL). *MPS* malaria parasites, *HIV* human immunodeficiency virus. ^a^Pearson’s Chi-squared test; ^b^Student’s *t* test ^c^Fisher’s exact test for count data; ^d^Fisher’s exact test for count data with simulated *P*-value (based on 5e + 05 replicates)

### Linkage disequilibrium

**Fig. 1:**
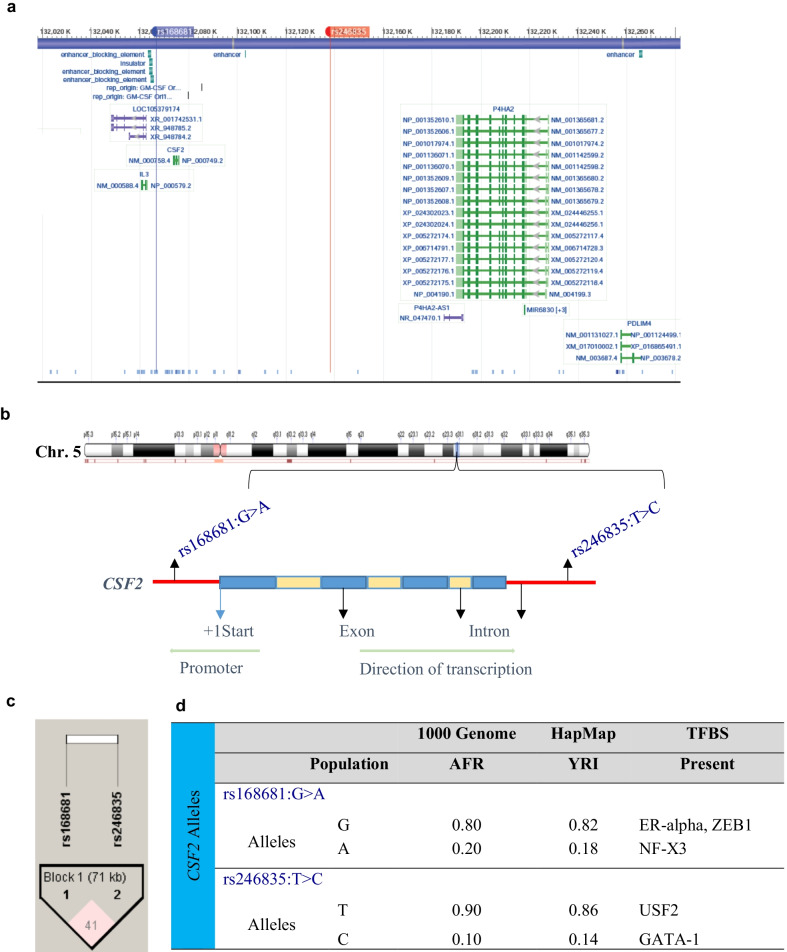
*CSF2* (5q31.1) region, transcription factor binding sites and linkage disequilibrium. **a** and **b** Location of the *CSF2* region on chromosome 5q31.1. *CSF2* is 9379 bp and is composed of four exons. Chromosome position (build GRCh38.p13) for the selected SNPs under investigation; rs168681:G > A is located at 132066757, rs246835:T > C located at 132138332 [58]. **c** Linkage disequilibrium between the selected *CSF2* SNPs rs168681:G > A and rs246835:T > C (*D*′ = 0.416, LOD = 4.32, *r*^2^ = 0.01). **d** *CSF2* genotypes allele frequencies from the International HapMap project for Yoruba (YRI; Nigeria). Allele frequencies from the 1000 Genome project shows African (AFR) ancestry. Transcription factor binding analyses of the *CSF2* gene variants. *AFR* African, *YRI* Yoruba, *ER-alpha* estrogen receptor alpha, *ZEB1* zinc finger E-box binding homeobox 1, *NF-X3* nuclear factor I-X3, *USF2* upstream transcription factor 2, *GATA* GATA binding protein-1

The location of the two SNPs selected for investigation in the *CSF2* gene region (5q31.1) [58] is shown in Fig. 1a and b. The chromosomal location of rs168681:G > A is 5:132066757, 7032 bp upstream of TSS of *CSF2*, and 1279 bp downstream of LOC108449898. The chromosomal location of rs246835:T > C SNP is 5:132138332, 64544 bp downstream of the TSS of *CSF2*, 34642 bp downstream of LOC112997558, and 46544 bp upstream of the human *P4HA2-AS1* gene [58] (Fig. 1a, b).

Linkage disequilibrium analysis revealed a weak association between the two *CSF2* variants (*D*′ = 0.416, LOD = 4.32, *r*^2^ = 0.01; Fig. 1c). Departures from linkage equilibrium (LE) were significant for the overall sample and stratified by aparasitemic, non-SMA, and SMA groups. However, the departure from LE was not significant in the SMA group after multiple testing, suggesting that these departures are marginal and only identified as significant due to the large sample size.

### Selection of *CSF2* SNPs

As recently described [50], a pilot high-throughput genotyping experiment was performed using the Human Immunochip^®^ (coated with > 196 K markers, Illumina^®^, San Diego, CA, USA) in a subset of children representing “polarized extremes” of severe and non-severe malaria: cases (SMA with Hb < 5.0 g/dL, n = 70) *vs.* controls (non-SMA with Hb > 8.0 g/dL, n = 74). Immunochip^®^ data were analyzed using logistic regression analysis, with an additive mode of inheritance and identified an intergenic SNP rs246835:T > C within the *CSF2* haploblock that was associated with increased risk to SMA [odds ratio (OR) = 2.771, 95% confidence interval (CI) = 0.904–8.494, *P* = 0.059]. We selected rs246835:T > C since this SNP is part of a potential distal enhancer with the loss of TFBS for USF2 and creation of TFBS for GATA-1 by switch of T allele to C allele (Fig. 1d). rs246835:T > C had a minor allele frequency (MAF) of 0.10 in the African (AFR) population (1000 Genome Project), and a MAF of 0.14 in the Yoruban (YRI) population (HapMap) (Fig. 1d). Though not in the Immunochip^®^, SNP rs168681:G > A had desirable characteristics for further exploration, including: (1) a MAF of 0.20 (1000 Genome Project) in the AFR population and a MAF of 0.18 in the YRI population (HapMap), and (2) potential functional effects, where the wild-type G allele creates TFBS for ER-alpha and ZEB1, and transition to the minor A allele ablates such binding and creating a TFBS for NF-X3. These TFBSs are part of a potential distal enhancers (Fig. 1d).

### Distribution of the *CSF2* genotypes, haplotypes, and diplotypes

Following genotyping, SNP calling was successful for rs168681:G > A and rs246835:T > C variants, with both genotypes present in 1203 out of 1654 study participants recruited into cohorts 1 and 2. To increase the power of analysis, no imputations were performed to infer genotypes, haplotypes or diplotypes. As such, only data with both SNPS present for each participant were utilized for subsequent statistical analyses (Table 2).

**Table 2:** Distribution of *CSF2* genotypes, haplotypes, and diplotypes

Variants	Total	Aparasitemic (MPS negative)	Non-SMA (Hb ≥ 5.0 g/dL)	SMA (Hb < 5.0 g/dL)	*P* value
No. of participants	1203	250	772	181	
*CSF2:*g.-7032 G > A (rs168681:G > A) genotype					
GG	636 (52.87)	134 (53.60)	407 (52.72)	95 (52.49)	0.9972^a^
GA	476 (39.57)	98 (39.20)	305 (39.51)	73 (40.33)	
AA	91 (7.56)	18 (7.20)	60 (7.77)	13 (7.18)	
* P* value	0.8847	1.0000	0.7869	1.0000	
Allele frequency (p/q)	0.727/0.273	0.732/0.268	0.725/0.275	0.726/0.274	
HWE, *P* value	0.8847	1.0000	0.7869	1.0000	
*CSF2:*g.64544T > C (rs246835:T > C) genotype					
TT	984 (81.80)	196 (78.40)	636 (82.38)	152 (83.98)	0.2202^a^
TC	125 (10.39)	35 (14.00)	77 (9.97)	13 (7.18)	
CC	94 (7.81)	19 (7.60)	59 (7.64)	16 (8.84)	
* P* value	2.6077 × 10^–55^	2.0014 × 10^–09^	1.9886 × 10^–36^	2.6515 × 10^–13^	
Allele frequency (p/q)	0.870/0.130	0.854/0.146	0.874/0.126	0.876/0.124	
HWE, *P* value	2.6182 × 10^–55^	2.0014 × 10^–9^	1.9896 × 10^–36^	2.6528 × 10^–13^	
*CSF2:*g.-7032 G > A (rs168681:G > A)/*CSF2:*g.64544T > C (rs246835:T > C) haplotypes					
GT	1470 (61.10)	297 (59.40)	949 (61.50)	224 (61.90)	0.5565^a^
GC	278 (11.60)	69 (13.80)	170 (11.00)	39 (10.80)	
AT	623 (25.90)	130 (26)	400 (25.90)	93 (25.70)	
AC	35 (1.50)	4 (0.80)	25 (1.60)	6 (1.70)	
LE, *P* value	1.6781 × 10^–13^	1.2543 × 10^–06^	2.0835 × 10^–07^	0.0307	
*CSF2:*g.-7032 G > A (rs168681:G > A)/*CSF2:*g.64544T > C (rs246835:T > C) diplotypes					
GT/GT	500 (41.56)	93 (37.20)	331 (42.88)	76 (41.99)	0.3255^a^
GT/GC	71 (5.90)	25 (10.00)	38 (4.92)	8 (4.42)	
GC/GC	65 (5.40)	16 (6.40)	38 (4.92)	11 (6.08)	
GT/AT	399 (33.17)	86 (34.40)	249 (32.25)	64 (35.36)	
GT/AC	27 (2.24)	3 (1.20)	19 (2.46)	5 (2.76)	
GC/AC	85 (7.07)	17 (6.80)	56 (7.25)	12 (6.63)	
AT/AT	50 (4.16)	9 (3.60)	37 (4.79)	4 (2.21)	
AT/AC	4 (0.33)	1 (0.40)	2 (0.26)	1 (0.55)	
AC/AC	2 (0.17)	0 (0.00)	2 (0.26)	0 (0.00)	

Data are presented as proportions [*n*, (%)] unless otherwise stated. Children (*n* = 1203) were categorized into aparasitemic controls (*n* = 250; no parasitemia) and according to the WHO definition of SMA [53], into either non-SMA (*n* = 772; Hb ≥ 5.0 g/dL) or SMA (*n* = 181; Hb < 5.0 g/dL). ^a^Fisher’s exact test with simulated *P*-values. *MPS* malaria parasites, *Hb* hemoglobin, *CSF2* colony-stimulating factor 2, *p* major allele, *q* minor allele, *HWE* Hardy–Weinberg Equilibrium, *LE* linkage equilibrium

The distribution of *CSF2* genotypes, haplotypes, and diplotypes in aparasitemic, non-SMA, and SMA groups is presented in Table 2. The genotypic proportions for rs168681:G > A (*P* = 0.9972), and rs246835:T > C (*P* = 0.2202) were comparable across the groups. The allele frequencies for the study population is comparable to those from the International HapMap Project and 1000 Genomes Project (Fig. 1d). The HWE analyses showed a departure for the rs246835:T > C for the overall population, and in the aparasitemic, non-SMA, and SMA groups. There was no departure from HWE for the rs168681:G > A polymorphic variant (Table 2). The distribution of haplotypes (*P* = 0.5565), and diplotypes (*P* = 0.3255) were not significantly different across the study groups.

### Relationship between *CSF2* variants and the rate of malaria episodes

In malaria holoendemic regions, such as western Kenya, children experience multiple episodes of malaria from infancy onward, with occasional life-threatening complications of SMA before the development of naturally acquired malaria immunity [7]. We, therefore, investigated the impact of the *CSF2* variants on malaria episodes over a follow-up period of 36 months. Factors associated with the rate of malaria episodes were determined using a generalized linear model (Poisson regression). Results for the covariates that emerged for the 6029 recorded malaria episodes over 36 months of follow-up are shown in Table 3. Age at enrollment was an important variable for the rate of malaria episodes, with older children presenting a decreased risk over time [incidence rate ratio (IRR; for a delta of 1 month) = 0.967 (95% CI 0.963–0.970), *P* < 0.0001)]. Children who were HIV-1 positive had a lower incidence rate of malaria episodes (*P* = 0.0023), relative to HIV-1 negative participants, as did female study participants (*P* = 0.0013), compared to their male counterparts. Carriage of the homozygous double deletion (-α^3.7^/-α^3.7^) for α^+^-thalassemia also conferred a protective effect against malaria episodes (*P* = 0.0068) relative to homozygous αα/αα carriers. Protection against multiple bouts of malaria over time was also associated with co-inheritance of homozygosity for the sickle cell disease (Hb SS, *P* < 0.0001) and heterozygosity for the sickle cell trait [Hb AS, (*P* < 0.0001). Protective effects against the longitudinal risk of malaria were also present in heterozygous carriers of the rs246835 TC genotype (*P* = 0.0277) relative to the wild type genotype TT. Similarly, the positional interaction of the *CSF2* AC/GC diplotype was associated with decreased malaria episodes (*P* = 0.0015) compared to wild-type diplotype GT/GT. Conversely, co-inheritance of the *CSF2* AT/GC diplotype increased the rate of malaria episodes (*P* = 0.0237). There was also a trend towards more malaria episodes in carriers of the *CSF2* GC/GT diplotype, however, the trend was not significant (*P* = 0.0663) (Table 3).

**Table 3:** Rate of malaria and SMA episodes

Variable names	Event	Baseline	Estimate	Std. error	*z* value	IRR (95% CI)	*P* value
Malaria episodes							
(Intercept)			− 1.389	0.032	− 43.218	0.249 (0.234–0.265)	< 0.0001
Age at enrollment			− 0.034	0.002	− 18.123	0.967 (0.963–0.970)	< 0.0001
Cohort*	499	576	0.044	0.029	1.509	1.045 (0.987–1.106)	0.1313
HIV1 (+)	36	1039	− 0.318	0.104	− 3.044	0.728 (0.593–0.893)	0.0023
Sex (female)	540	535	− 0.089	0.028	− 3.225	0.915 (0.867–0.966)	0.0013
-α^3.7^/-α^3.7^	215	860	− 0.097	0.036	− 2.709	0.908 (0.846–0.974)	0.0068
Hb SS	14	1061	− 0.750	0.181	− 4.155	0.473 (0.332–0.673)	< 0.0001
Hb AS	159	916	− 0.228	0.042	− 5.455	0.796 (0.734–0.864)	< 0.0001
* CSF2:g.*64544 TC genotype	115	960	− 1.102	0.501	− 2.201	0.332 (0.125–0.886)	0.0277
* CSF2* GC/GT diplotype	66	1009	0.926	0.504	1.837	2.525 (0.940–6.787)	0.0663
* CSF2* AT/GC diplotype	46	1029	1.142	0.505	2.262	3.132 (1.164–8.422)	0.0237
* CSF2* AC/GC diplotype	25	1050	− 0.313	0.099	− 3.174	0.732 (0.603–0.887)	0.0015
Model fit							
AIC			5509.852				
Deviance			2254.5387	1063			
Null deviance			2743.8236	1074			
SMA episodes							
(Intercept)			− 4.147	0.132	− 31.452	0.016 (0.012–0.021)	< 0.0001
Age at enrollment			− 0.056	0.009	− 6.118	0.946 (0.929–0.963)	< 0.0001
Cohort*	499	576	− 0.441	0.137	− 3.222	0.644 (0.492–0.841)	0.0013
HIV 1 (+)	36	1039	1.060	0.239	4.431	2.887 (1.806–4.614)	< 0.0001
Hb SS	14	1061	1.034	0.341	3.031	2.813 (1.441–5.492)	0.0024
Hb AS	159	916	− 0.673	0.223	− 3.017	0.510 (0.329–0.790)	0.0026
* CSF2* AT haplotype	485	590	0.291	0.122	2.396	1.338 (1.055–1.699)	0.0166
Model fit							
AIC			1360.7875				
Deviance			861.3021	1068			
Null deviance			972.6857	1074			

Poisson regression model fit was determined using Akaike information criterion, with the heuristic approach being performed on an iterative manner to exclude potentially irrelevant variables. Data are ranked per variables as follows; the intercept, followed by metric variables (age), categorical variables (cohort, HIV1), genetic variables (sex, -α^3.7^/-α^3.7^, Hb SS, Hb AS) and *CSF2* gene variants. *Cohort presented in the table were patients recruited into the study in the 2007–2012 study period. IRR confidence intervals with GLM were computed using the delta method. *Std. Error* standard error, *IRR* incidence rate ratio, *95% CI* 95% confidence interval, *HIV 1* human immunodeficiency virus 1, *-α*^*3.7*^*/-α*^*3.7*^ α^+^-thalassemia homozygous mutant, *Hb SS* sickle cell diseases, *Hb AS* sickle cell trait, *CSF2* colony-stimulating factor 2, *AIC* Akaike information criterion

### Relationship between *CSF2* variants and the rate of SMA episodes

The effect of the *CSF2* variants on the rate of SMA episodes over a follow-up period of 36 months was investigated using a Poisson regression as described above. Factors associated with the rate of SMA for the 297 episodes that occurred over the 36 months of follow-up are shown in Table 3. Consistent with the results observed for susceptibility to malaria, children who were older at admission into the study had decreased incidence rates for SMA (*P* < 0.0001). Similarly, children who were recruited into cohort 2 (2007–2012) had lower incidences of SMA (*P* = 0.0013), relative to those enrolled in study cohort 1 (2003–2005). Unlike the protection observed against malaria in HIV-1-positive children, being positive for HIV-1 markedly increased the risk of SMA (*P* < 0.0001). Inheritance of the double mutation that confers sickle cell disease (Hb SS) increased the rate of SMA (*P* = 0.0024), whereas carriage of sickle cell trait (Hb AS) protected against SMA episodes (*P* = 0.0026). The only *CSF2* variant found to influence the rate of SMA episodes was carriage of the *CSF2* AT haplotype, which increased the risk of SMA episodes (*P* = 0.0166) compared to the wild-type GT haplotype.

### Impact of *CSF2* variants on the longitudinal risk of malaria infections

After determining the impact of *CSF2* variants on the rate of malaria and SMA episodes, we then determined the effect of the variants on the time between events using an ordered-events, generalized Cox proportional hazard model. Shown in Table 4 are the covariates that emerged from the model. Children who were older upon enrollment had a reduced hazard risk (HR) for malaria (*P* < 0.0001). Although retained in the model, cohort (*P* = 0.1159), HIV-1 positivity (*P* = 0.1711), sex (*P* = 0.0580) and -α^3.7^/-α^3.7^ (*P* = 0.0874) were not significant predictors for the time between malaria infections (Table 4). However, inheritance of either the Hb SS mutant genotype (*P* < 0.0001) or the Hb AS variant (*P* = 0.0001) decreased the longitudinal hazard for malaria infections. Carriage of the *CSF2* AC haplotype was also protective against malaria infections (*P* = 0.0045) relative to wild-type haplotype (GT), while co-inheritance of the *CSF2* GC/GT diplotype showed a non-significant trend towards a lower risk of malaria (*P* = 0.1108) compared to wild type (GT/GT) (Table 4).

**Table 4:** Longitudinal risk of malaria and SMA infections and all-cause mortality

Variable names	Event	Baseline	Coefficient	Std. Error^a^	*z* value	HR (95% CI)	HR inverse (95% CI)	*P* value
Malaria infections								
Age at first hospital visit			− 0.041	0.003	− 14.865	0.960 (0.956–0.964)	1.042 (1.036–1.047)	< 0.0001
Cohort*	498	575	0.071	0.045	1.572	1.074 (1.014–1.137)	0.931 (0.853–1.018)	0.1159
HIV 1 (+)	36	1037	− 0.196	0.143	− 1.369	0.822 (0.670–1.009)	1.217 (0.919–1.611)	0.1711
Sex (female)	540	533	− 0.082	0.043	− 1.896	0.921 (0.873–0.972)	1.086 (0.997–1.182)	0.0580
-α^3.7^/-α^3.7^	215	858	− 0.092	0.054	− 1.709	0.912 (0.850–0.978)	1.097 (0.987–1.219)	0.0874
Hb SS	14	1059	− 0.726	0.174	− 4.163	0.484 (0.340–0.689)	2.066 (1.468–2.908)	< 0.0001
Hb AS	159	914	− 0.242	0.063	− 3.858	0.785 (0.723–0.852)	1.274 (1.127–1.441)	0.0001
* CSF2* AC haplotype	30	1043	− 0.325	0.114	− 2.840	0.723 (0.606–0.863)	1.384 (1.106–1.731)	0.0045
* CSF2* GC/GT diplotype	66	1007	− 0.153	0.096	− 1.595	0.859 (0.756–0.975)	1.165 (0.966–1.405)	0.1108
Model fit								
Test			Test stat	DF	*P* value	Number of total visits (*N*)	13,253	
LR test			587.8063	9	< 0.0001	Number of malaria events	5299	
Score test			539.2203	9	< 0.0001	AIC	68,758.3586	
Wald test			273.68	9	< 0.0001	Concordance (Std. Error)	0.6175 (0.0059)	
SMA infections								
Age at first hospital visit			− 0.047	0.010	− 4.587	0.954 (0.936–0.973)	1.048 (1.027–1.070)	< 0.0001
Cohort*	498	575	− 0.426	0.146	− 2.915	0.653 (0.494–0.863)	1.532 (1.150–2.040)	0.0036
HIV 1 (+)	36	1037	1.050	0.253	4.147	2.858 (1.761–4.640)	0.350 (0.213–0.575)	< 0.0001
Hb SS	14	1059	0.989	0.467	2.118	2.688 (1.322–5.465)	0.372 (0.149–0.929)	0.0342
Hb AS	159	914	− 0.706	0.302	− 2.335	0.494 (0.309–0.789)	2.026 (1.120–3.665)	0.0196
* CSF2* AT haplotype	485	588	0.232	0.133	1.745	1.262 (0.982–1.621)	0.793 (0.611–1.029)	0.0809
Model fit								
Test			Test stat	DF	*P* value	Number of total visits	13,022	
LR test			81.9523	6	< 0.0001	Number of SMA events	248	
Score test			83.5002	6	< 0.0001	AIC	3306.7994	
Wald test			66.77	6	< 0.0001	Concordance (Std. Error)	0.6814 (0.0171)	
All-cause mortality								
Age at enrollment			− 0.070	0.022	− 3.173	0.932 (0.893–0.974)	1.0728 (1.027–1.120)	0.0015
HIV 1 (+)	36	1038	2.801	0.320	8.763	16.452 (8.794–30.779)	0.061 (0.033–0.114)	< 0.0001
Hb SS	14	1060	1.882	0.605	3.110	6.568 (2.005–21.509)	0.152 (0.047–0.499)	0.0019
Hb AS	158	916	− 1.216	0.600	− 2.026	0.296 (0.091–0.961)	3.373 (1.041–10.937)	0.0427
* CSF2:*g.-7032 GA genotype	436	638	0.632	0.294	2.150	1.881 (1.058–3.345)	0.532 (0.299–0.946)	0.0315
* CSF2* GC/GC diplotype	56	1018	0.945	0.501	1.887	2.574 (0.964–6.869)	0.389 (0.146–1.037)	0.0592
Model fit								
Test			Test stat	DF	*P* value			
LR test			71.1327	6	< 0.0001			
Score test			150.5798	6	< 0.0001	AIC	671.609	
Wald test			87.77	6	< 0.0001	Concordance (Std. Error)	0.7598 (0.0334)	

Independent increments according to Anderson–Gill method were used for ordered multiple-outcome-per-subject Cox proportional hazard model fit to investigate on the time-to-event of covariates for malaria and SMA infections. Additionally, the COX proportional hazard model was used to predict all-cause mortality outcomes. A positive coefficient indicates a worse prognosis, whereas a negative coefficient indicates a better prognosis. Data are ranked per variables as follows; the metric variables (age at first hospital visit), followed by categorical variables (cohort, HIV 1), genetic variables (sex, -α^3.7^/-α^3.7^, Hb SS, Hb AS) and *CSF2* genetic variants. ^a^Standard error robust estimate. *Cohort presented in the table are patients recruited into the study in the 2007–2012 study period. The sample size (*N* = 13,253) consisted of all visits of N1 = 1073 (= event + baseline) patients retained for this analysis. *Std. Error* standard error, *HR* hazard ratio, *HR inverse* reciprocal of hazard ratio, *95% CI* 95% confidence interval, *HIV 1* human immunodeficiency virus 1, *-α*^*3.7*^*/-α*^*3.7*^ α^+^-thalassemia homozygous mutant, *Hb SS* sickle cell diseases, *Hb AS* sickle cell trait, *CSF2* colony-stimulating factor 2, *AIC* Akaike information criterion, *LR test* likelihood ratio test

### Impact of *CSF2* variants on the longitudinal risk of SMA infections

Cox proportional hazard model was also used to examine the influence of *CSF2* variants rs168681:G > A and rs246835:T > C on the time-to-event for malaria infections that culminated in the development of SMA (Table 4). Consistent with the Poisson models examining the rates of SMA, time-to-event modeling revealed that older children (at enrollment) had a lower risk of SMA (*P* < 0.0001, i.e., the hazard decreases by a factor of 0.954 for each additional month of enrollment age) as did children enrolled in cohort 2 (*P* = 0.0036). Co-infection with malaria and HIV-1 was a strong predictor of increased susceptibility to SMA (*P* < 0.0001), as was carriage of the Hb SS genotype (*P* = 0.0342). Conversely, inheritance of the Hb AS variant decreased the longitudinal risk of SMA (*P* = 0.0196). Although non-significant, there was a trend towards increased risk of SMA in children with the *CSF2* AT haplotype (*P* = 0.0809).

### Influence of *CSF2* variants on the longitudinal risk of all-cause mortality

The influence of covariates on the risk of all-cause mortality was investigated using a Cox proportional hazard model (Table 4). Older children (at enrollment) had a reduced risk of mortality (*P* = 0.0015), whereas children with HIV-1 had a 16-times higher risk of mortality (*P* < 0.0001). Children with Hb SS also had a marked increase in the risk of mortality (*P* = 0.0019). Conversely, carriage of sickle cell trait (Hb AS) conferred a lower hazard risk for mortality (*P* = 0.0427). Inheritance of the rs168681 GA genotype significantly increased the risk of dying during the follow-up period (*P* = 0.0315), as did carriage of the *CSF2* GC/GC diplotype, although not significant (*P* = 0.0592).

## Discussion

Advances in human gene mapping, along with an increased understanding of the molecular mechanisms of protective immunity, illustrate that susceptibility to malaria and its clinical outcomes is conditioned by genotypic variation [8]. The work presented here is focused on gaining an improved understanding of the molecular basis of clinical immunity to SMA in pediatric populations living under intense *P. falciparum* transmission [8]. In such holoendemic regions, clinical immunity to malaria is mediated, at least in part, by the progressive generation of antibodies following repeated malaria infections [7, 24]. However, prior to the development of naturally acquired malarial immunity, children are reliant upon innate immune responses and can experience life-threatening complications that include severe anemia, hyperparasitemia, and respiratory distress [7, 8]. To better understand the pathogenesis of SMA, and as a consequence, reduce mortality, our studies primarily focus on genes and gene pathways involved in innate immunity. Consistent with this strategy, the current study investigated the influence of genetic variants flanking *CSF2* (rs168681:G > A and rs246835:T > C) on the rate and timing of malaria and SMA over a 36-month longitudinal follow-up period during the developmental phase of naturally acquired malarial immunity. The impact of *CSF2* gene variants on all-cause mortality was also investigated. Since other factors can influence the development of malaria, SMA, and mortality, we also determined the influence of important covariates in all statistical models.

Previous studies from our laboratories have shown that the host releases both pro- and anti-inflammatory cytokines, chemokines, growth factors, and effector molecules as part of the innate immune response to malaria infections [7, 8]. Among the growth factors is GM-CSF, which is highest among children with uncomplicated malaria relative to those with non-SMA and SMA [24]. However, children with SMA have higher levels of circulating GM-CSF than individuals with non-SMA, suggesting a complicated pattern of production during acute infection [24]. Studies by others have shown divergent results on clinical outcomes in malaria. For example, elevated GM-CSF levels are associated with severe malaria complications (i.e., splenomegaly and leukocytosis) in some investigations, while others have found a protective role for GM-CSF [59]. Recent studies suggest that the production of TLR 7/8-driven GM-CSF in cord blood is an independent predictor of enhanced malaria risk over the first year of life, suggesting that GM-CSF indeed plays an important role in malarial immunity [21]. To expand knowledge on the role of GM-CSF in malaria, we examined the impact of genetic variations around *CSF2* on susceptibility to malaria and its severe disease manifestations (i.e., SMA and mortality) in children as they progressively develop immunity to clinical malaria.

Selection of the polymorphic variants was based on interrogating SNPs (and their combinations) that are previously unexplored in malaria and have the potential to impart functional changes on GM-CSF production. For example, the presence of the wild-type G allele at the rs168681:G > A locus within one distal enhancer produce TFBSs for ER-alpha and ZEB1, whereas transition to the A allele creates a binding site for NF-X3. Variation at rs246835:T > C locus within another distal enhancer of *CSF2* has a TFBS for USF2 in the presence of the T allele and a TFBS for GATA-1 in carriers of the C allele. Previous studies have confirmed that *CSF* contains consensus elements for ZEB1 and GATA [27, 28]. Since transcription factors are adaptor molecules that detect regulatory sequences in the DNA and target the assembly of protein complexes that control gene expression, mutations within the transcription factor binding sites (TFBS) can alter gene expression [60]. The allele frequencies for the two SNPs in the study cohort were comparable to those in the Yoruba population (HapMap Project) and the African population (1000 Genome Project), suggesting that the two loci have been steadily maintained in ethnic groups of African descent [61]. The rs168681:G > A variant displayed HWE, whereas the rs246835:T > C locus had a significant departure from HWE in the overall population, and in each of the clinical groups investigated. Although a departure from HWE at rs246835:T > C locus could be attributed to historical pressure from malaria [62], there were comparable frequencies of the genotypes, as well as haplotypes and diplotypes in combination with rs168681:G > A across the clinical groups (i.e., aparasitemic, non-SMA, and SMA), suggesting an influence of a large sample size. This finding suggests that the two variants selected for investigation are likely not under strong selection pressure. The LD measures for the two loci with a *D*′ = 0.416 and *r*^2^ = 0.01 indicate that the two variants are not strongly linked [63].

Analysis of the interacting covariates revealed that the children’s age, cohort (2003–2005 vs 2007–2012), sex (female vs male), and HIV-1 status (+ vs −) altered the incidences and risk of malaria and SMA over the longitudinal follow-up of 36 months. Further, co-inheritance of genetic variants; α^+^-Thalassemia and/or sickle cell traits influenced longitudinal susceptibility to malaria and SMA infections and mortality outcomes. These results parallel those of previous studies that reported variability in the degree of protection conferred by hemoglobinopathies against mild and severe malaria (reviewed in [64]). In addition, the selected *CSF2* homozygous and heterozygous variants (genotypes, haplotypes, and diplotypes) altered the incidence rate and hazard ratios to malaria and SMA over the follow-up period. Results presented here complement previous studies illustrating that children living in regions with high transmission rates for *P. falciparum* experience multiple infections prior to developing malarial immunity, with the greatest burden of severe disease manifesting in children less than 5 years of age [8, 50–52]. As such, longitudinal studies have a greater ability to detect genetic factors, and covariates that can influence both the rate and timing of repeated malaria episodes and the development of severe disease over time.

A Poisson regression model estimating the rate of malaria episodes over 36 months revealed that the presence of the rs246835 TC heterozygote genotype and *CSF2* AC/GC diplotype decreased the incidence of malaria, while the *CSF2* AT/GC diplotype increased the incidence of malaria episodes. Additional Poisson analysis identified significantly more episodes of SMA among children who inherited the *CSF2* AT haplotype. To further elucidate the impact of the *CSF2* genetic variants on the longitudinal risk of malaria and SMA, a Cox proportional hazard model was fit with independent increments according to the Anderson–Gill method. Carriage of the *CSF2* AC haplotype was associated with a lower hazard risk for malaria infections. Additionally, inheritance of the *CSF2* AT haplotype that significantly increased the incidence rate for SMA was associated with an elevated longitudinal hazard for SMA, albeit non-significant. Results obtained in this study support our previous findings that showed innate immune response genes influence longitudinal susceptibility to malaria and subsequent development of SMA in this *P. falciparum* holoendemic region [50–52]. Moreover, results presented here illustrate that *CSF2* genetic variants are associated with both the rate and timing of malaria and SMA episodes during the development of naturally acquired malarial immunity in children living in a high malaria transmission region. Although transcript and protein levels in circulation for CSF2/GM-CSF were not measured in the current study due to sample availability, we hypothesize that the variants alter the gene products and, thereby, influence susceptibility to malaria and SMA, as demonstrated in our previous studies for other genetic variants [50, 61].

We also investigated predictors of all-cause mortality across 36 months using Cox proportional hazard modeling and found that younger children and those with HIV-1 had an increased risk of death. In addition, carriage of the Hb AS trait protected against mortality, whereas inheritance of the Hb SS homozygous mutant imparted 6.5 times higher risk of dying. Several *CSF2* variants were also associated with increased susceptibility to all-cause mortality, including carriage of the rs168681 GA genotype (1.8 times higher) and the *CSF2* GC/GC diplotype (2.6 times higher). Interestingly, neither of these variants significantly impacted susceptibility to either malaria or SMA, suggesting that their impact on mortality may be due to non-malaria-related causes.

## Conclusions

In summary, although the two variants investigated correspond to distal enhancer regions within the 5q31.1, and are associated with altered susceptibility to malaria, SMA, and all-cause mortality, the underlying molecular mechanism(s) for the disease associations remain largely unknown. To our knowledge, this is the first study examining the impact of two genetic variants flanking the 5q31.1 gene region on longitudinal malaria disease outcomes and all-cause mortality. It will be important for future studies to establish the precise role of *CSF2*, and the soluble inflammatory mediator it encodes, GM-CSF, on susceptibility to malaria, and the subsequent development of severe disease once an individual becomes infected.

## Data Availability

All data generated or analyzed during this study are included in this published article [and its additional information files].
